# Supplementary material for: Spatial-proteomics reveals phospho-signaling dynamics at subcellular resolution
Source: Nat Commun. 2021 Dec 7;12:7113. doi: 10.1038/s41467-021-27398-y (PMC8651693; doi:10.1038/s41467-021-27398-y)
Supplement: Supplementary file 1 — Supplementary information [file 41467_2021_27398_MOESM1_ESM.pdf]

Supplementary material for:

Spatial-proteomics reveals phospho-signaling dynamics at subcellular resolution

Ana Martinez-Val[1], Dorte B. Bekker-Jensen[1,2], Sophia Steigerwald[1,3], Claire Koening[1], Ole Østergaard[1], Adi Mehta[4], Trung Tran[4], Krzysztof Sikorski[4],Estefanía Torres-Vega[5], Ewa Kwasniewicz[6], Sólveig Hlín Brynjólfsdóttir[7], Lisa B. Frankel[7], Rasmus Kjøbsted[8], Nicolai Krogh[9], Alicia Lundby[1,5], Simon Bekker-Jensen[6], Fridtjof Lund-Johansen[4,*], Jesper V. Olsen[1,*]

[1] NovoNordisk Foundation Center for Protein Research, Proteomics Program, Faculty of Health and Medical Sciences, University of Copenhagen, Copenhagen, Denmark

[2] Evosep Systems, Odense, Denmark

[3] Max Planck Institute of Biochemistry, Department of Proteomics and Signal Transduction, Martinsried, Germany

[4] Department of Immunology, Oslo University Hospital, Rikshospitalet, Postboks 4950, Nydalen, 0424 Oslo, Norway

[5] Cardiac Proteomics, Faculty of Health and Medical Sciences, University of Copenhagen, Copenhagen, Denmark

[6] Center for Healthy Aging, Department of Cellular and Molecular Medicine, University of Copenhagen, Copenhagen, Denmark

[7] Danish Cancer Society, Copenhagen, Denmark

[8] Department of Nutrition, Exercise and Sports, University of Copenhagen, Copenhagen, Denmark.

[9] Department of Cellular and Molecular Medicine, University of Copenhagen, Copenhagen, Denmark

* Correspondence to: jesper.olsen@cpr.ku.dk; fridtjol@gmail.com

- Supplementary Table 1.
- Supplementary Figures 1-12.
- Supplementary Notes 1-2.
- Supplementary References.

Supplementary Table 1. Comparative table different MS-based subcellular fractionation protocols.

	Digitonin + Homogeneization+ Ultracentrifugation	hyperLOPIT: equilibrium density gradient ultracentrifugation			LOPIT-DC: differential centrifugation	Chemical fractionation
As shown in	Orre *et al*	Christoforou *et al*	Thul *et al*	Geladaki et al	Geladaki et al	Current Study
PMID	30609389	26754106	28495876	30659192	30659192	-
Cell line(s)	A431<br>U251<br>MCF7<br>NCI-H322<br>HCC-827	Mouse pluripotent stem cells (E14TG2a)	U2OS	U2OS	U2OS	HeLa<br>U2OS
Input	1x P15 (3x p10 A431)	~100 million cells	~300 million cells	~280 milliion cells	~70 million cells	1x P15 (~20e6 cells)
# Subcellular Fractions	5 fractions (FS1, FP1, FP2, FP3, FS2)	8 fractions from density gradient (out of 20) + cytosol + chromatin	20 fractions from density gradient + cytosol+chromatin		10 fractions	6 fractions
Labeling	TMT 10-plex	TMT 10-plex			TMT 10-plex	-
Offline Fractionation	HiRIEF (2x72 fractions)	High-pH reverse phase chromatography (24 fractions)			High-pH reverse phase chromatography (18-22 fractions)	-
Data acquisition method	DDA	DDA (SPS-MS3)			DDA (SPS-MS3)	DIA
LC Gradient Duration	90 min	120 min			120 min	21 min
MS Acquisition time (per replicate)	9 days	~2-3 days (per TMT10plex experiment)			1.5 days	2.5 h

Supplementary Figure 1: High-throughput subcellular fractionation shows specific subcellular compartments enriched in each fraction.

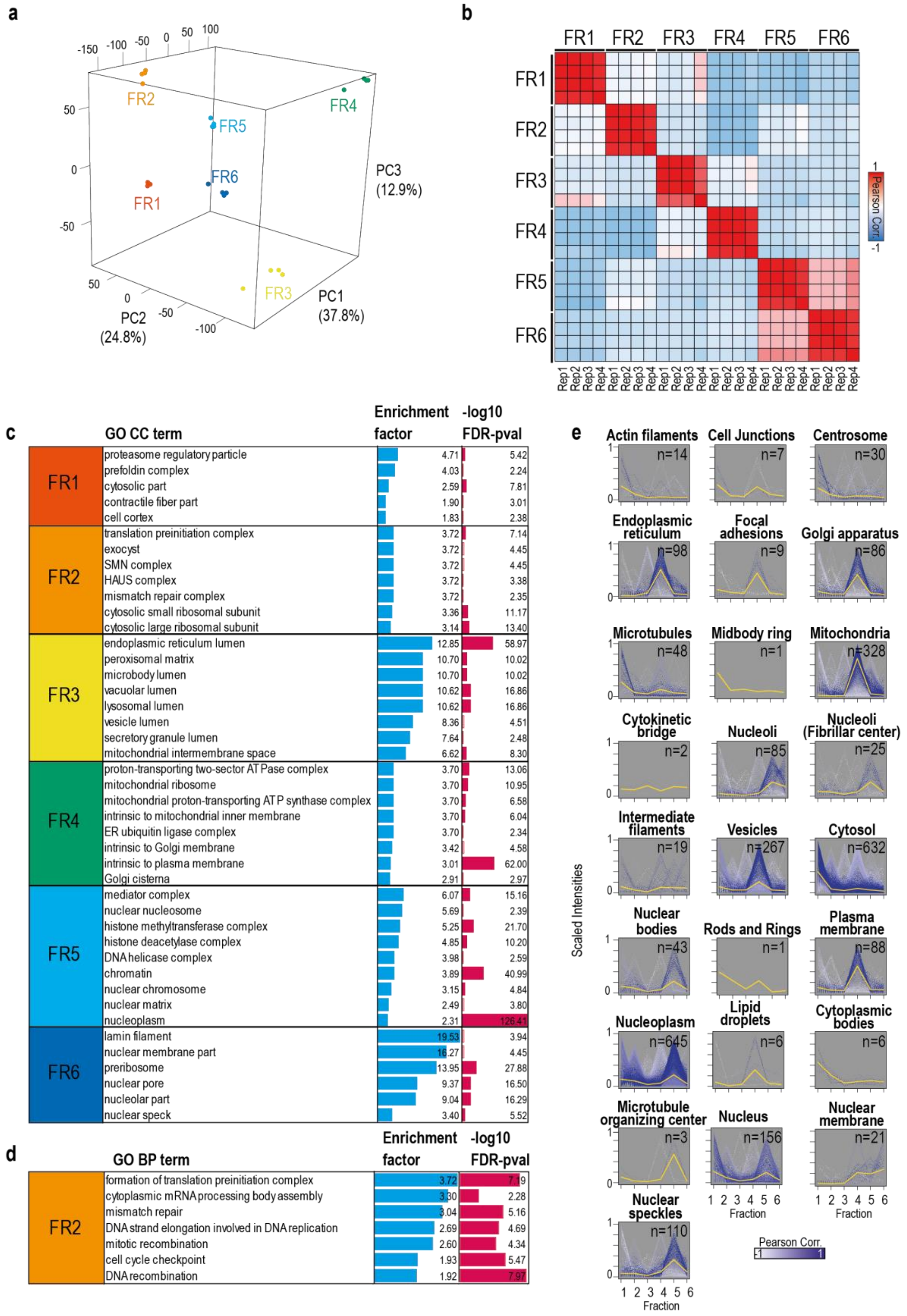

(A) Principal Component Analysis of fractions obtained from the subcellular fractionation protocol applied to HeLa cells (n=4 replicates).

(B) Correlation plot showing Pearson correlation values between HeLa subcellular fractions for full proteome samples.

(C) Gene Ontology Cell Compartment terms enriched (Fisher Exact test, two-sided) in the clusters of proteins more abundant in each fraction.

(D) Gene Ontology Biological Process terms enriched (Fisher Exact test, two-sided) in the clusters of proteins more abundant in each fraction.

(E) Profile-plots of cell compartment markers obtained from The Cell Atlas[1] in the subcellular proteome dataset. Scaled intensity across fractions is plotted for each independent replicate. Gradient of white to blue indicates Pearson correlation to the centroid of each distribution, which is highlighted as a yellow line.

Supplementary Figure 2: Dual distribution of Endoplasmic Reticulum and Lysosome markers.

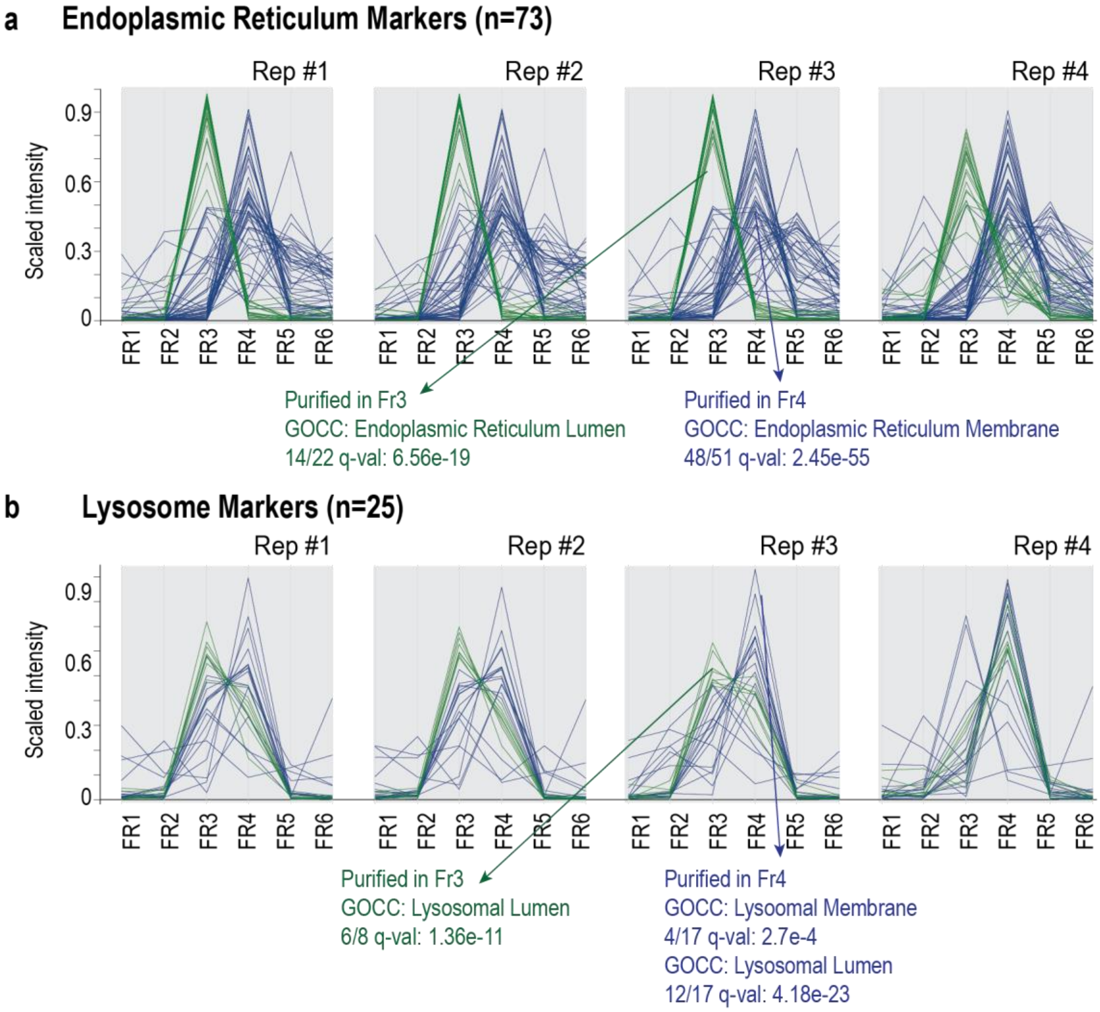

(A) Profile plots of protein markers for the endoplasmic reticulum for each independent replicate in HeLa cells.

(B) Profile plots of protein markers for the Golgi apparatus for each independent replicate in HeLa cells.

Supplementary Figure 3: Reproducible subcellular fractionation in two different cell lines.

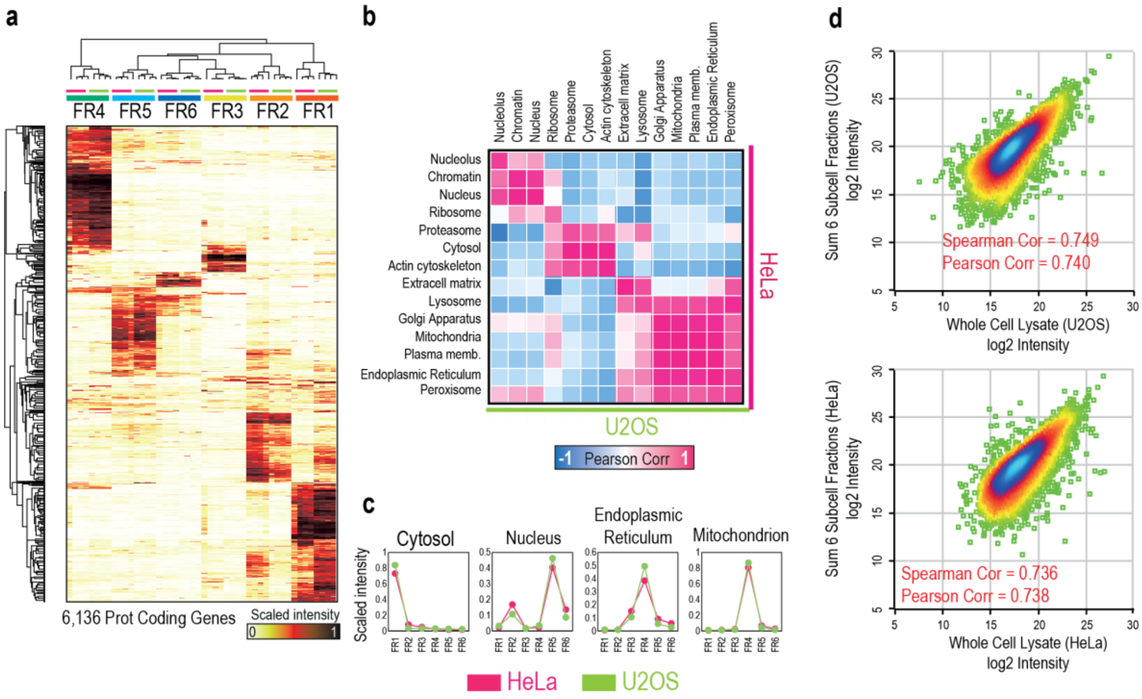

(A) Heatmap of protein scaled intensities across fractions in the HeLa (pink) and U2OS (green) subcellular proteome dataset.

(B) Correlation plot of the centroids of the distribution of cellular compartment markers between HeLa and U2OS datasets.

(C) Plot of centroids (measure as average per fraction of four replicates) of relevant cellular compartments in HeLa (pink) and U2OS (green). Source Data is provided as a Source Data file.

(D) Scatter-plot of whole cell lysate log2 protein intensities (as average of 4 replicates) against sum of log2 protein intensity of six subcellular fractions (as average of 4 replicates) obtained for U2OS cell line (top) and HeLa cell line (bottom).

Supplementary Figure 4: Evaluation of protein loss during washes.

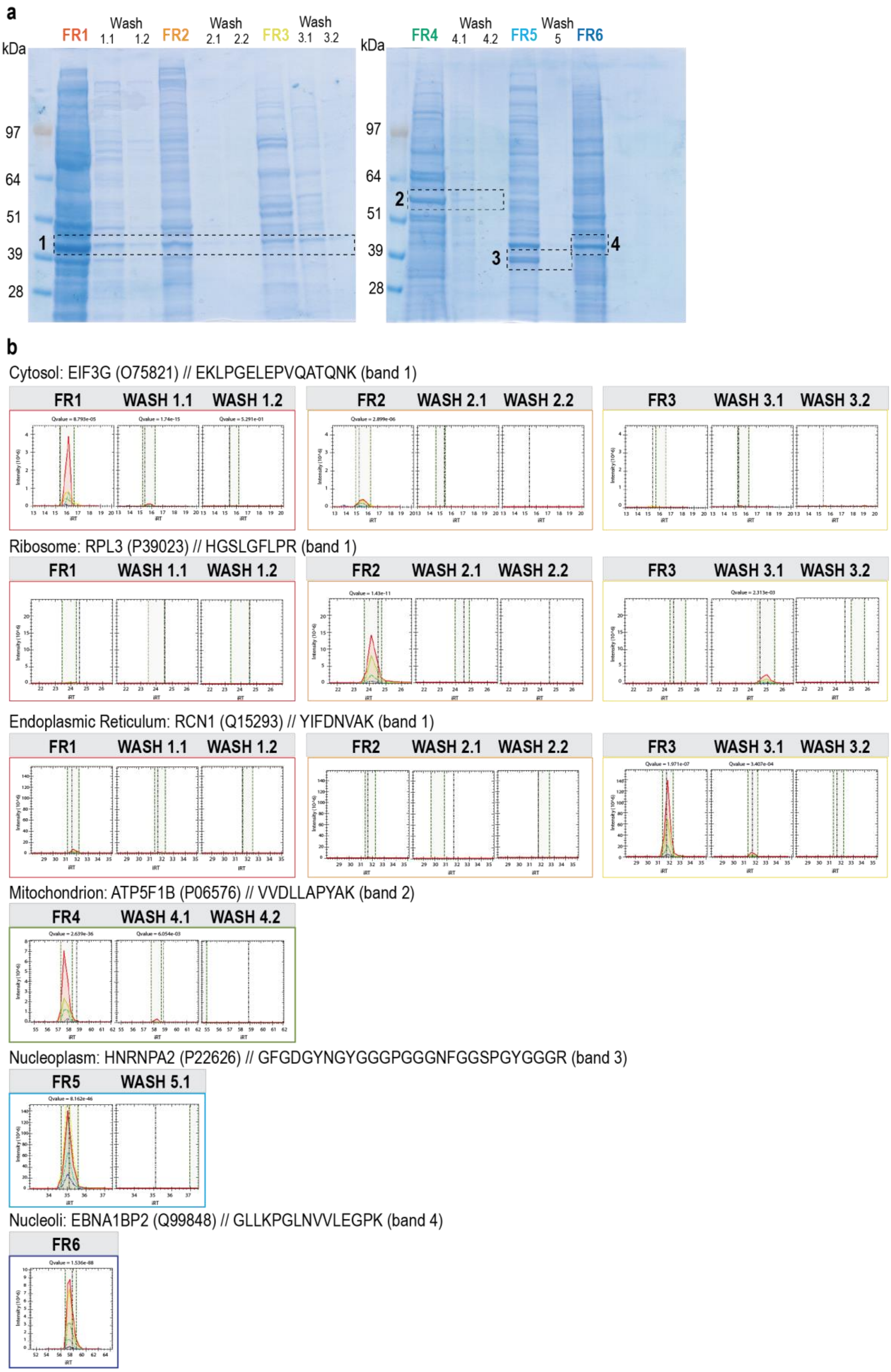

(A) SDS-PAGE gels, stained with InstantBlue^{TM} Coomassie Protein Stain. Half of the total volume for each fraction and wash was loaded. The gel bands cut for LC-MS/MS analysis are marked in dashed boxes. Experiment was performed in duplicates.

(B) Extracted ion chromatograms (MS1) for relevant markers for each organelle or compartment purified in the different fractions and washes.

Supplementary Figure 5: Evaluation of the subcellular phospho-proteome.

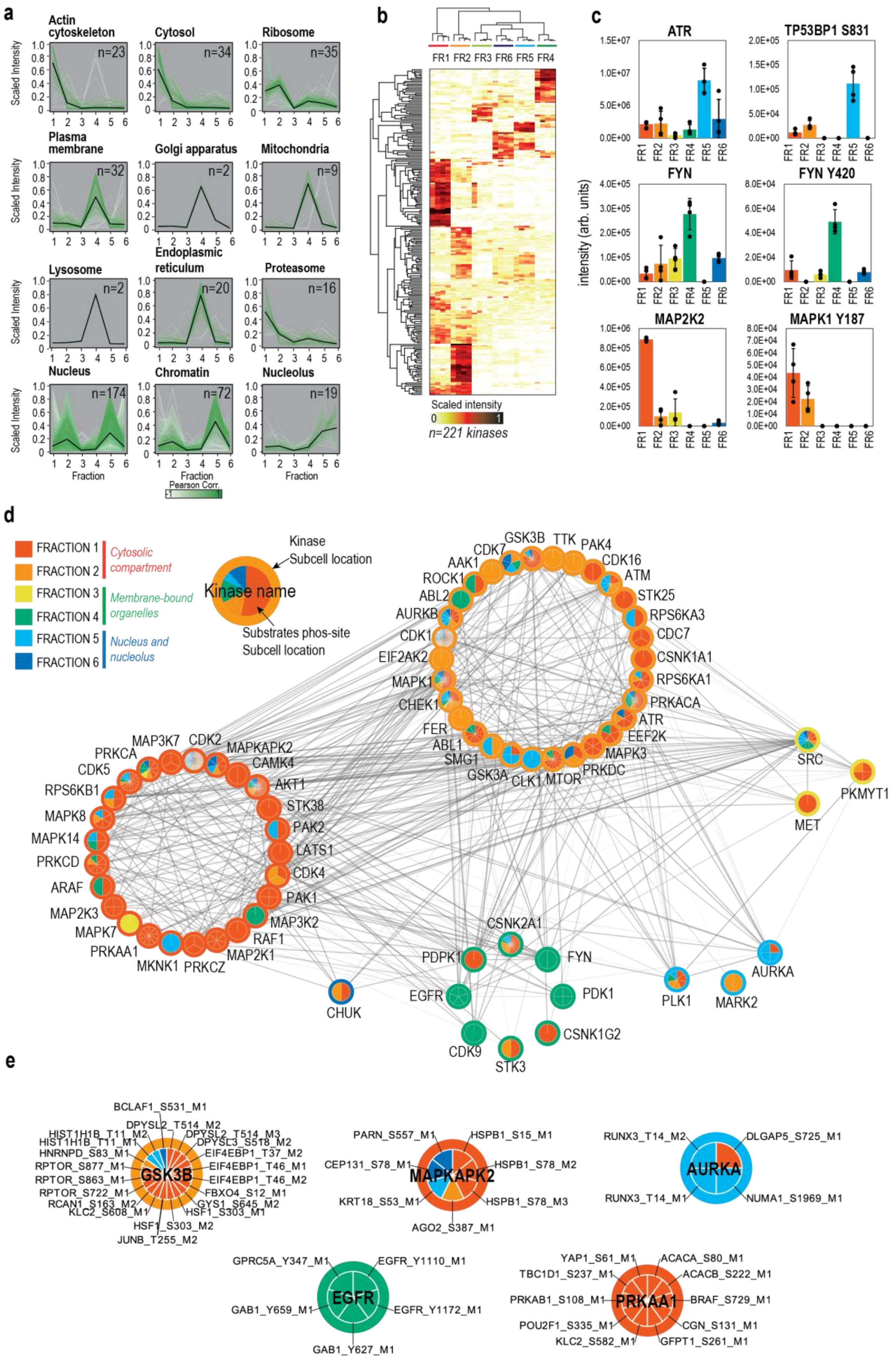

(A) Profile-plots of cell compartment markers in the subcellular phospho-proteome HeLa dataset. Scaled intensity across fractions is plotted for each independent replicate. Gradient of white to green indicates Pearson correlation to the centroid of each distribution, which is highlighted as a black line.

(B) Heatmap of protein scaled intensities across fractions of the kinases present in the HeLa subcellular proteome dataset.

(C) Bar-plot of intensities across fractions in the HeLa subcellular fractionation datasets corresponding to protein kinases and representative phosphorylation substrates. Height of the bars represents the mean protein intensity of n=4 experimental replicates, and error bars represent the standard deviation. Source Data is provided as a Source Data file.

(D) Network map of kinases and associated substrates (annotated from PhosphoSitePlus[2]). Kinases are grouped in circles by their main subcellular location, which is indicated by the corresponding color in the outer circle of the node. Within each node, each substrate is represented in a pie chart, where the color also indicates its main subcellular location.

(E) Examples of kinases and substrates from the network in D.

Supplementary Figure 6: Metamass analysis of published datasets.

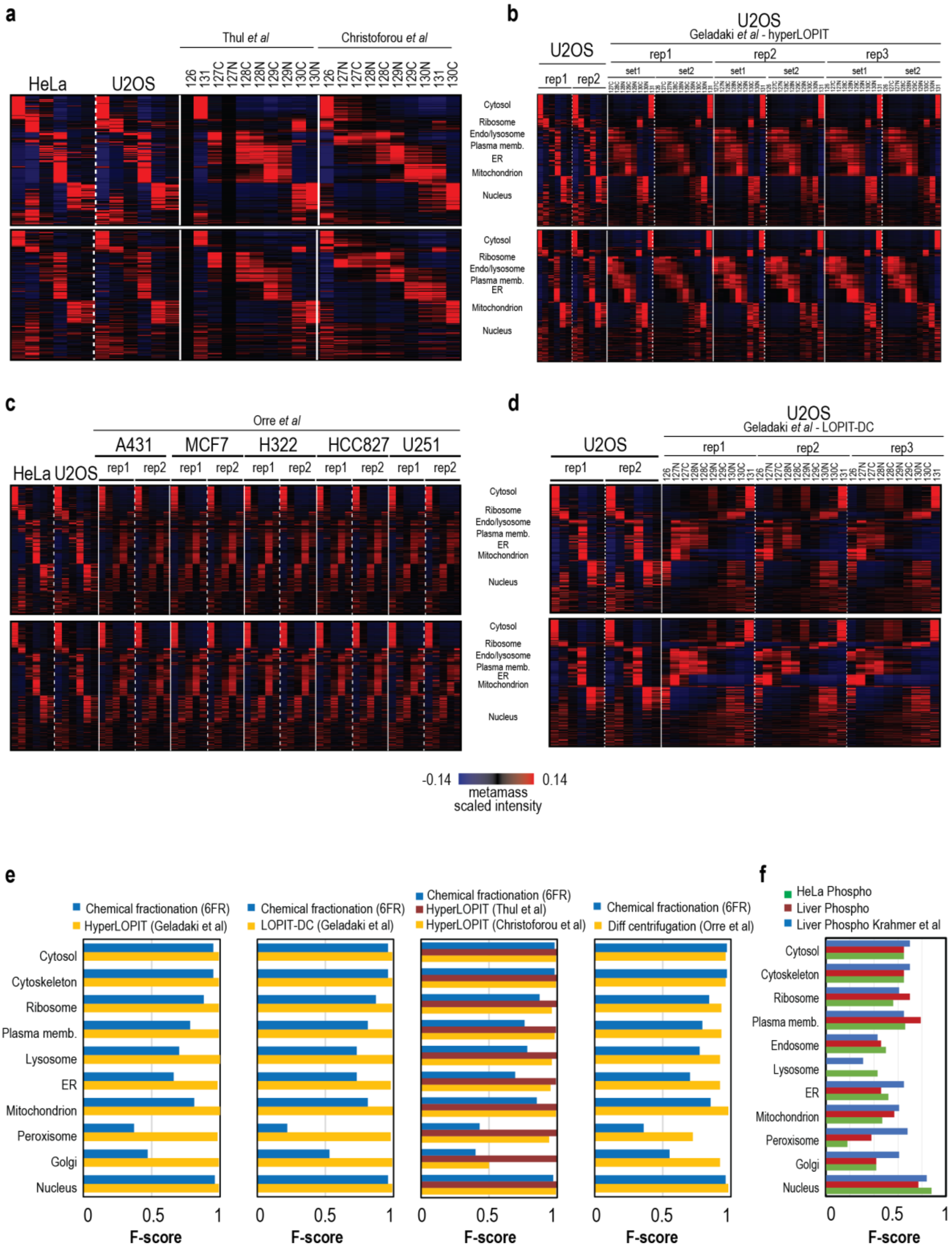

(A-D) Heatmaps showing protein distribution across fractions obtained from HeLa and/or U2OS using the present subcellular fractionation protocol and for other published studies. Proteins were classified and sorted using the Excel-based analysis tool MetaMass (Suppl. Data 4). All heatmaps were obtained after normalizing gene distribution and center samples by mean in Cluster 3.0, and plotted in TreeView. For all heatmaps, top heatmap corresponds to protein classification based on the data from this study, and bottom heatmap corresponds to protein classifications based on each corresponding published study.

(A) Comparison of protein distribution across fractions obtained from HeLa and/or U2OS using the present subcellular fractionation and HyperLOPIT subcellular fractionation method used in Christoforou *et al*[3] and Thul *et al*[1].

(B) Comparison of protein distribution across fractions obtained from U2OS, either using the present subcellular fractionation and HyperLOPIT subcellular fractionation method used in Geladaki *et al*[4].

(C) Comparison of protein distribution across fractions obtained from HeLa and U2OS using the present subcellular fractionation and the different cell lines used in Orre *et al*[5].

(D) Comparison of protein distribution across fractions obtained from U2OS, either using the present subcellular fractionation or LOPIT-DC subcellular fractionation method used in Geladaki *et al*[4].

(E) F-score barplots for the protein assignment to organelles in the present study (blue) and different subcellular fractionation published studies (yellow and red).

(F) F-score barplots for the phosphosite assignment to organelles in the present study (green – HeLa and red – Liver) and Krahmer et al[6] (blue).

Supplementary Figure 7: Protein translocation in response to EGF stimulation.

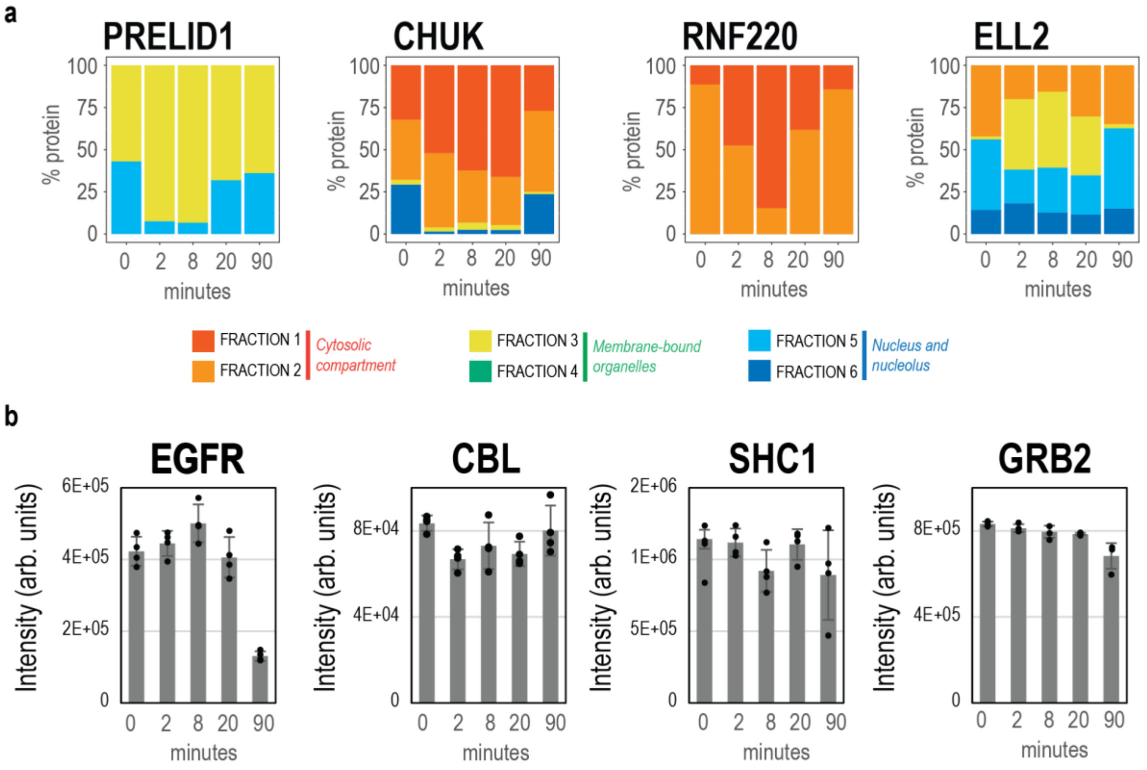

(A) Stacked bar plots of scaled intensity per fraction of significant proteins found to be significantly moving between compartments, which were identified in the translocation analysis in figure 4B.

(B) Bar plots of mean protein intensity of EGFR, CBL, SHC1 and GRB2 proteins at different time points upon stimulation with EGF. Data correspond to a full proteome quantitative experiment on HeLa cells treated with EGF at 2, 8, 20 and 90 minutes. Experiment were performed in quadruplicates. Height of the bars represents the mean protein intensity of n=4 replicates, and error bars represent the standard deviation.

Source Data is provided as a Source Data file.

Supplementary Figure 8: Subcellular fractionation applied to frozen tissues.

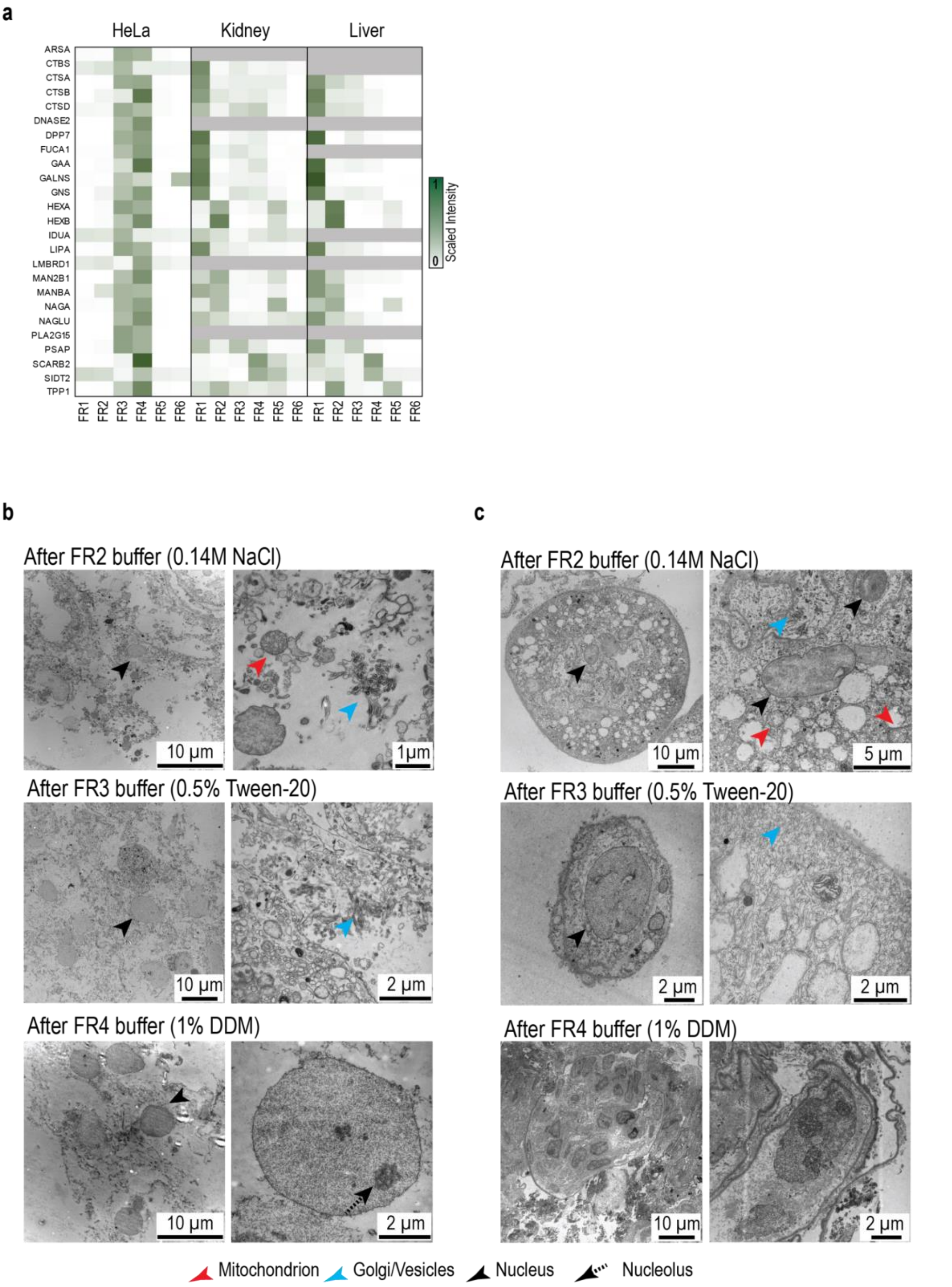

(A) Heatmap of scaled intensities across fractions for lysosome markers in the HeLa, Kidney and Liver datasets.

(B) Representative images from Transmission Electron Microscopy of liver samples at different stages of the subcellular fractionation protocol. Red arrows point to

mitochondria, blue arrows point to the Golgi apparatus, black arrows point to the nucleus and black dotted arrows point to the nucleoli.

(C) Representative images from Transmission Electron Microscopy of kidney samples at different stages of the subcellular fractionation protocol. Red arrows point to mitochondria, blue arrows point to the Golgi apparatus, black arrows point to the nucleus and black dotted arrows point to the nucleoli.

Sample preparation was performed in technical duplicates derived from the same organ, which were then pooled for TEM acquisition of each subcellular fractionation step.

Supplementary Figure 9: Molecular response at subcellular level in U2OS cells after osmotic shock.

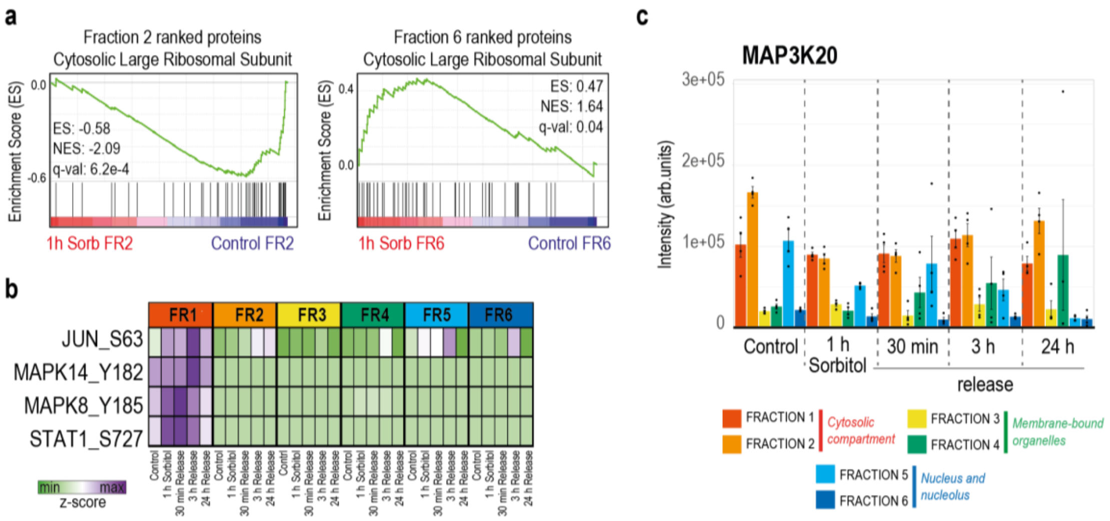

(A) GSEA plots for the GOCC term "Cytosolic Large Ribosomal Subunit" obtained from the protein ratios (1 hour Sorbitol vs Control) in fraction 2 and fraction 6.

(B) Heatmap of phosphorylation site z-score intensities of JNK and p38 signaling targets.

(C) Bar plot of protein intensity across fractions and time points of MAP3K20. Height of the bars represents the mean intensity of n=4 measurements of the protein, and error bars represent the standard error of the mean. Source Data is provided as a Source Data file.

Supplementary Figure 10: validation of large ribosomal subunit translocation to the nucleoli after stimulation with sorbitol.

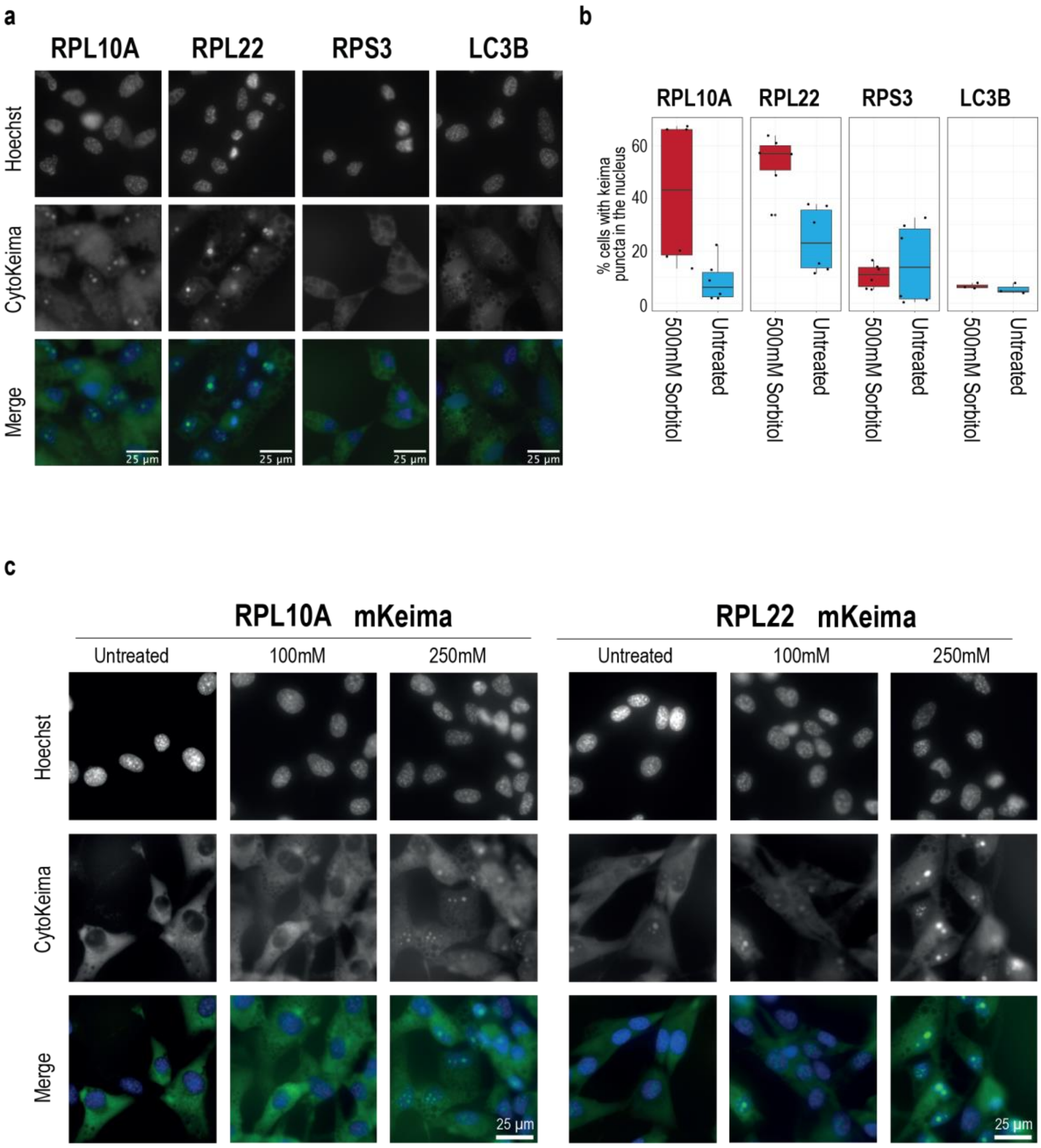

(A) Representative images of TIG3 cells expressing mKeima-tagged RPL10A, RPL22, RPS3 or LC3B and treated with 500mM sorbitol for 3h and analyzed for pH neutral keima signal (CytoKeima). Replicates for each experiment were as follows: RPL22 and LC3B n=4, RPL10Aa and RPS3 n=2.

(B) Quantification of percentage of cells with keima puncta in the nucleus for of TIG3 cells expressing mKeima-tagged RPL10A, RPL22, RPS3 or LC3B and treated with 500mM sorbitol for 3h and analyzed for pH neutral keima signal (CytoKeima). Quantification was performed in technical replicates: n=3 for LC3B and n=6 for RPL10A, RPL22 and RPS3. Boxplots show medians and limits indicate the 25th and 75th

percentiles, whiskers extend 1.5 times the interquartile range from the 25th and 75th percentiles, outliers are represented by dots. Source Data is provided as a Source Data file.

(C) Representative images of TIG3 cells expressing mKeima-tagged RPL10A untreated or treated with 100mM or 250 mM sorbitol for 3h and analyzed for pH neutral keima signal (CytoKeima). Experiment was performed for one biological replicate, imaging was performed in three technical replicates.

Supplementary Figure 11: rRNA processing changes after stimulation with sorbitol.

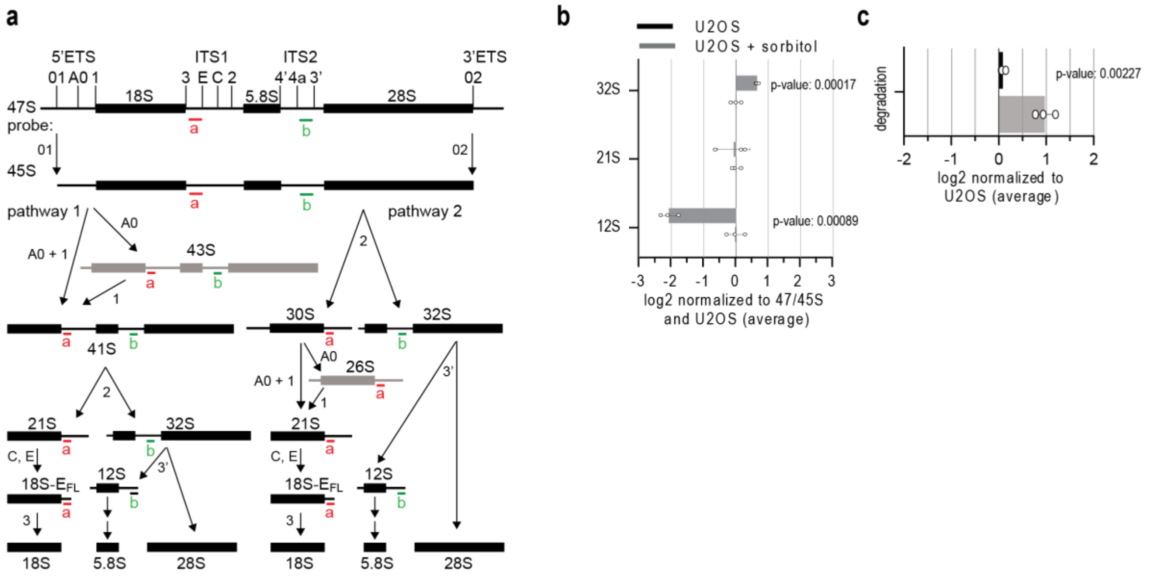

(A) Scheme of the human rRNA processing intermediates with annotated processing sites and a simplified outline of the two main processing pathways with short-lived precursors in grey. The position of probe a and b used in Figure 4F are in red and green, respectively.

(B) Quantification of a subset of rRNA intermediates from northern blot (n=3 replicates) in Figure 6F expressed as log2 fold change, internally normalized to 47/45S and the average of the three lanes containing RNA from control cells. Error bars indicate the standard deviation. Statistical significance was calculated with an unpaired t-test (two sided).

(C) Quantification of the area marked “degradation” in the right northern blot (n=3 replicates) in Figure 6F expressed as log2 fold change and normalized to the average of the three lanes containing RNA from control cells. Error bars indicate the standard deviation. Statistical significance was calculated with an unpaired t-test (two sided). Source Data for supplementary figures 11B and 11C are provided as a Source Data file.

Supplementary Figure 12: Translocation analysis workflow.

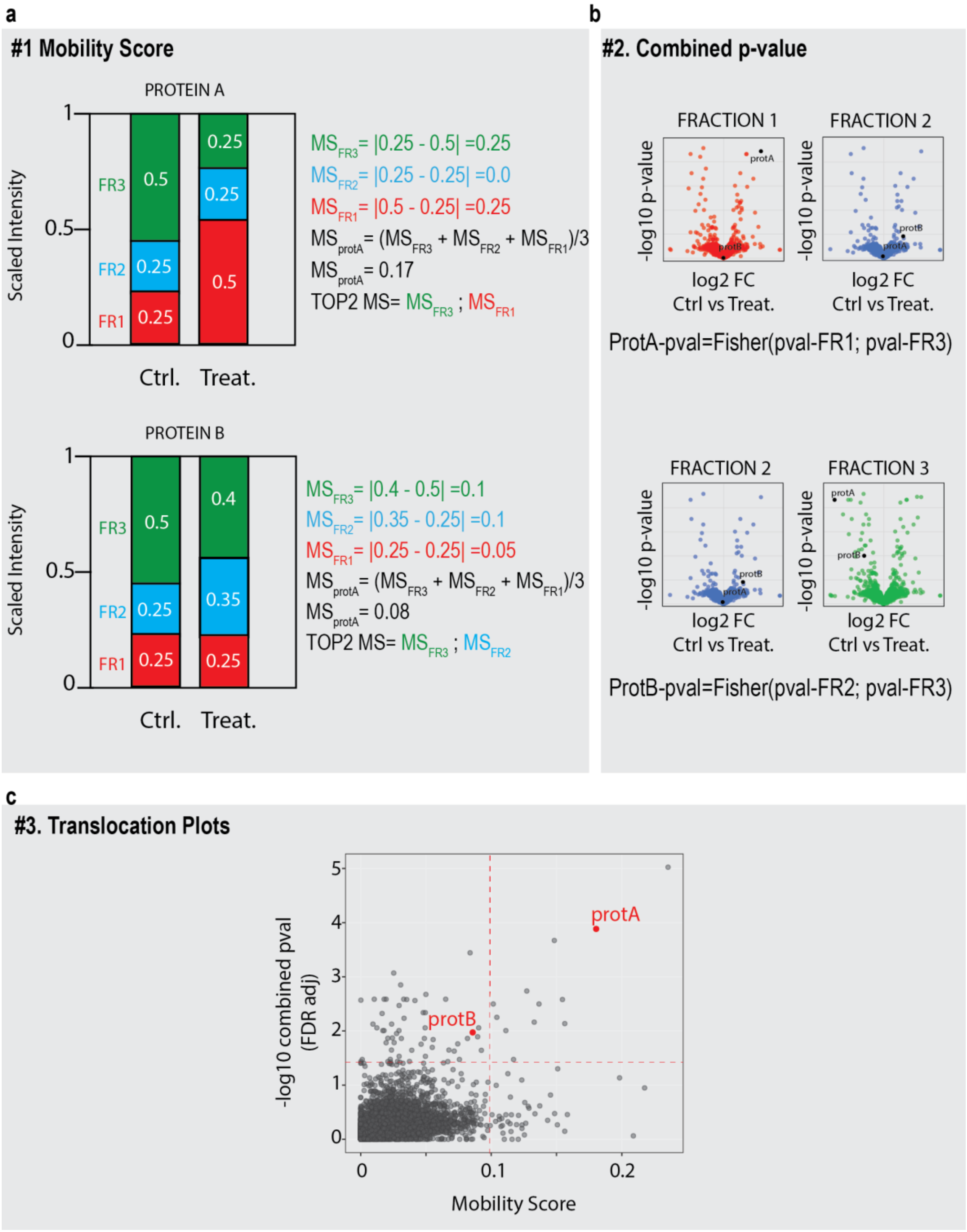

(A) Mobility Score calculation example.

(B) Combined p-value calculation example.

(C) Representation of the resulting translocation plot combining the mobility score and combined p-value (log10 transformed and adjusted for multiple comparisons)

Supplementary Note 1: assignment of proteins to dual or multiple locations.

In the main manuscript text, we state that 82% of the proteins were reproducibly identified in two or more of the six subcellular fractions. It has been published that a significant part of the proteome is not restricted to only one subcellular location[1]. However, it is very important to differentiate between identification in a subcellular fraction and actual co-localization of a protein in a subcellular niche. Merely identification cannot provide accurate information of the subcellular niche of the protein. In contrast, that information should be derived from the quantification of a protein's abundance across the six fractions, i.e. the relative enrichment of the protein in each fraction. Throughout the manuscript, we use the relative enrichment in each fraction to extrapolate the subcellular niches, assigning the fraction with the highest intensity as the main subcellular location for a protein.

When trying to accurately assess dual or multiple locations of proteins, our approach is limited by the fact this it is comprising only six fractions, which can group several subcellular niches. Methods that provide higher resolution, such as hyperLOPIT[3],LOPIT-DC[4] or SubCellBarCode[5], offer better insights in this regard. In fact, some bioinformatics tools have been developed to assess simultaneous protein sub-cellular localization in those datasets, such as the one described by Crook et al[7]. However, as already mentioned, the predictive outcome of this tool is better suited for subcellular approaches with more fractions analyzed.

Nevertheless, although with certain limitations due to the purification of only six subcellular compartments, our approach can also identify proteins that are present in multiple compartments simultaneously. In fact, we demonstrated in the main text the dual, and also dynamic, location of EGFR-adaptor proteins SHC1, GRB2 and CBL, which were all found in both the cytosol and the membrane-associated compartment (Main Figure 4C).

However, to assess if dual or multiple locating proteins are captured by our experimental approach, we investigated some proteins known to have dual localization according to the antibody-based fluorescent image analysis described in the publication by Thul et al[1]. As an example of proteins with dual/multiple location, Thul et al described CCAR1 and NDUFA9, which they found in both the nucleus and the Golgi apparatus or mitochondria, respectively. For both CCAR1 and NDUFA9, we find that in our dataset is in line with the observations by Thul et al as the majority of each protein is in FR5 (nucleoplasm) and FR4 (mitochondrion), respectively. Moreover, we can see some contribution of CCAR1 in FR4 (enriched in Golgi proteins), which is especially clear in

U2OS cells (see figure below). Similarly, for NDUFA9, we can see that the compartments with more presence of the protein after FR4 are those corresponding to the nuclear compartment (FR5 and FR6) (see figure below).

Moreover, in the Thul et al work, they also refer to ribosomal protein L19 as potentially present in the cytosol, the endoplasmic reticulum and the nucleoli. Same as before, we extracted the information for those proteins from our datasets, and found that those multiple location matches our quantitative data. When we plot the scaled intensity distribution of this protein across our six fractions, we can clearly see that it also in our datasets is distributed across those three subcellular compartments (see figure below).

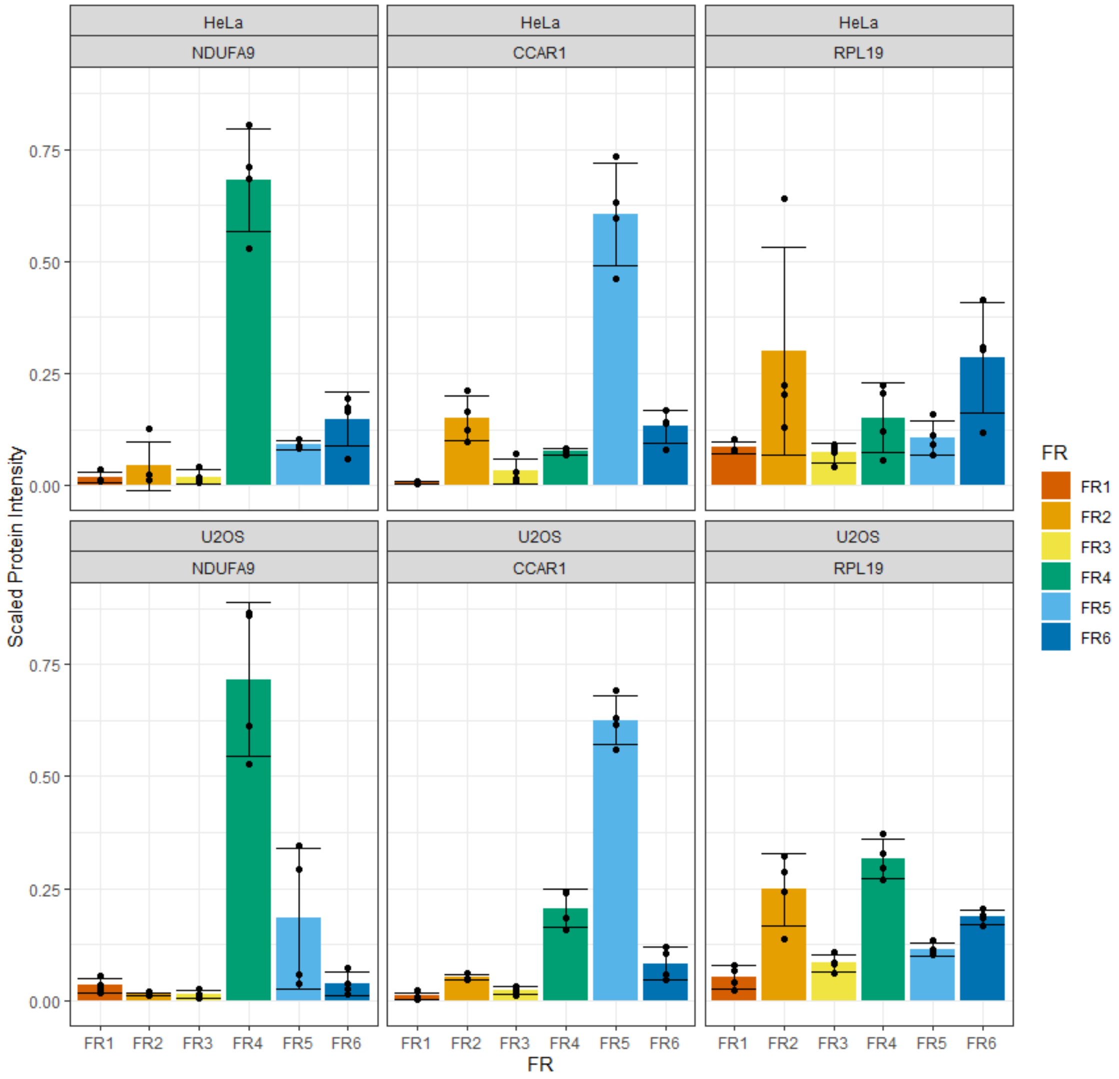

Barplot with scaled intensity across fractions of NDUFA9, CCAR1 and RPL19 in HeLa and U2OS. Height of the bars indicate the average of four replicates, and the error bars indicate the standard deviation of the measurements.

Supplementary Note 2: MetaMass II User Manual

Page 2: Overview
Page 3: Datasets
Page 4: Workbook for data output
Pages 5-7: K-means clustering
Pages 8-9: Comparing F-scores for two datasets
Pages 10-14: Classification of individual proteins
Pages 15-17 : Description of MetaMass functions
Pages 18: Scores from the Compartments Database

1

MetaMass II User Manual.

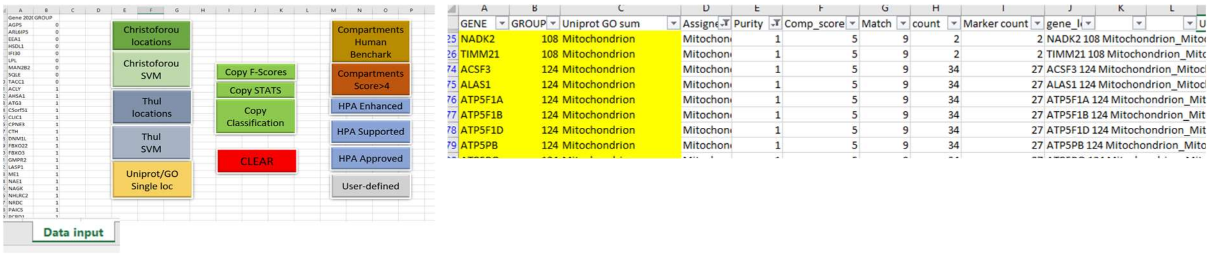

MetaMass II is a Macro-Enabled Excel Spreadsheet.

The input a list of protein identifiers and group-assignments obtained by cluster analysis (typically k-means clustering).

-Users paste the list into the spreadsheet and click buttons to select a set of markers for subcellular locations. The sets are from published articles or annotation databases.

The output is a list of subcellular locations for each protein, a score to indicate precision of the assigned location and statistics for the overall fit between the dataset and the marker set.

2

Data sets

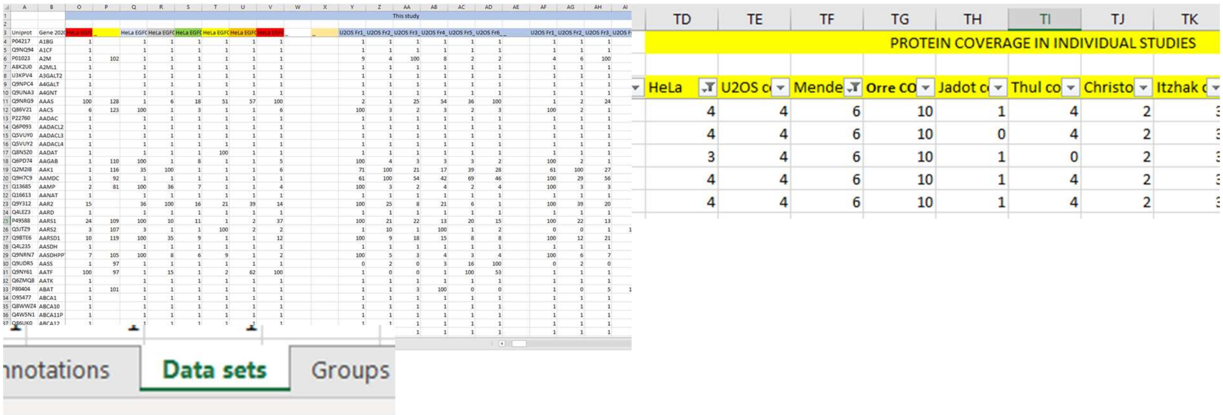

The Datasets sheet in MetaMass II contains normalized data from indicated studies.
Normalization to a fixed max value is recommended for better visualization of data in heatmaps.
Columns TD:TU can be used to filter the overlap between individual studies.

3

Workbook for data output

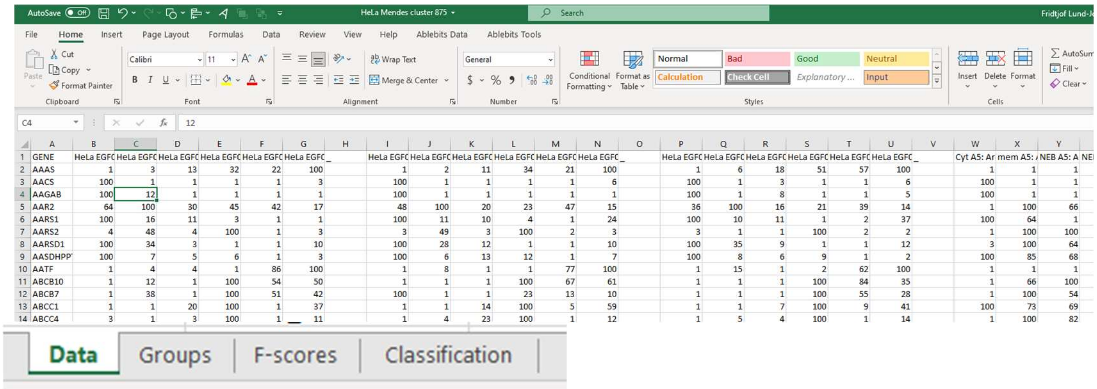

We recommend that MetaMass is used in combination with an Excel Workbook formatted for saving the output. Supplementary tables for individual comparisons can be used as templates. Here, we pasted the overlap between the HeLa results from this study and results from Mendes et al into the «Data» worksheet.

4

K-means clustering 1

A	B	C	D	E	F	G	H	I	J	K	L	M	N	O	P	Q	R	S	T	U	V
Gene 202(	HeLa EGF(	HeLa EGF(	HeLa EGF(	HeLa EGF(	HeLa EGF(	HeLa EGF(	_	HeLa EGF(	HeLa EGF(	HeLa EGF(	HeLa EGF(	HeLa EGF(	HeLa EGF(	_	HeLa EGF(	HeLa EGF(	HeLa EGF(	HeLa EGF(	HeLa EGF(	HeLa EGF0	3_FR6
AAAS	1	3	13	32	22	100		1	2	11	34	21	100		1	6	18	51	57	100	
AACS	100	1	1	1	1	3		100	1	1	1	1	6		100	1	3	1	1	6	
AAGAB	100	12	1	1	1	1		100	1	1	1	1	1		100	1	8	1	1	5	
AAR2	64	100	30	45	42	17		48	100	20	23	47	15		36	100	16	21	39	14	
AARS1	100	16	11	3	1	1		100	11	10	4	1	24		100	10	11	1	2	37	
AARS2	4	48	4	100	1	3		3	49	3	100	2	3		3	1	1	100	2	2	
AARSD1	100	34	3	1	1	10		100	28	12	1	1	10		100	35	9	1	1	12	
AASDHPP	100	7	5	6	1	3		100	6	13	12	1	7		100	8	6	9	1	2	
AATF	1	4	4	1	86	100		1	8	1	1	77	100		1	15	1	2	62	100	
ABCB10	1	12	1	100	54	50		1	1	1	100	67	61		1	1	1	100	84	35	
ABCB7	1	38	1	100	51	42		100	1	1	23	13	10		1	1	1	100	55	28	
ABCC1	1	1	20	100	1	37		1	1	14	100	5	59		1	1	7	100	9	41	
ABCC4	3	1	3	100	1	11		1	4	23	100	1	12		1	5	4	100	1	14	
ABCD3	2	1	2	100	43	39		1	5	3	100	66	69		1	1	2	100	83	39	

A	B	C	D	E	F	G	H	I	J	K	L	M	N	O	P	Q	R	S	T	U	V	W	X
Gene 202(	Cyt A5: Ar	mem A5: /	NEB A5: A	NEB-CBP /	PEB A5: Ar	_	Cyt B5: Ar	mem B5: /	NEB B5: A	NEB-CBP E	PEB B5: Ar	_	Cyt A5: Ar	mem A5: /	NEB A5: A	NEB-CBP /	PEB A5: Ar	_	Cyt B5: Ar	mem B5: /	NEB B5: A	NEB-CBP E	PEB B5: Area
AAAS	1	1	1	1	100		1	1	1	1	100		1	1	1	1	100		1	1	1	1	100
AACS	100	1	1	1	1		100	1	1	1	1		100	1	1	1	1		100	1	1	1	1
AAGAB	100	1	1	1	70		64	1	1	1	100		100	1	1	1	70		64	1	1	1	100
AAR2	1	100	66	1	93		1	43	100	1	47		1	100	66	1	93		1	43	100	1	47
AARS1	100	64	1	1	1		100	42	1	1	1		100	64	1	1	1		100	42	1	1	1
AARS2	1	100	100	1	1		1	100	21	1	1		1	100	100	1	1		1	100	21	1	1
AARSD1	3	100	64	1	1		100	10	21	1	1		3	100	64	1	1		100	10	21	1	1
AASDHPP	100	85	68	1	1		100	29	27	1	1		100	85	68	1	1		100	29	27	1	1
AATF	1	1	1	1	100		1	1	1	1	100		1	1	1	1	100		1	1	1	1	100
ABCB10	1	66	100	1	86		1	100	93	1	18		1	66	100	1	86		1	100	93	1	18
ABCB7	1	100	54	1	1		1	72	100	1	1		1	100	54	1	1		1	72	100	1	1

Results from the two studies were saved as separate tab-delimited text files for processing in Cluster 3.0. Both contain the first column with protein identifiers.

5

K-means clustering 2

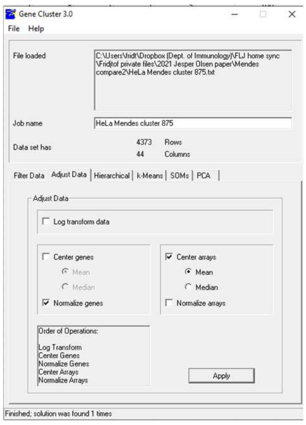

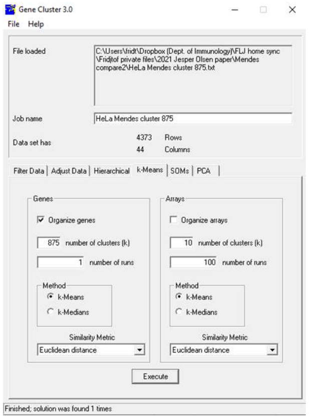

The Tab-delimited text files are opened in Cluster. 3.0, data and formatted as indicated above.
With 875 groups for k-means clustering, the groups will contain an average of five proteins. Larger groups will yield higher coverage and lower precision for assigning subcellular locations (see later).

6

The output from Cluster 3.0 is pasted into the «Groups» worksheet
in the Analysis Workbook

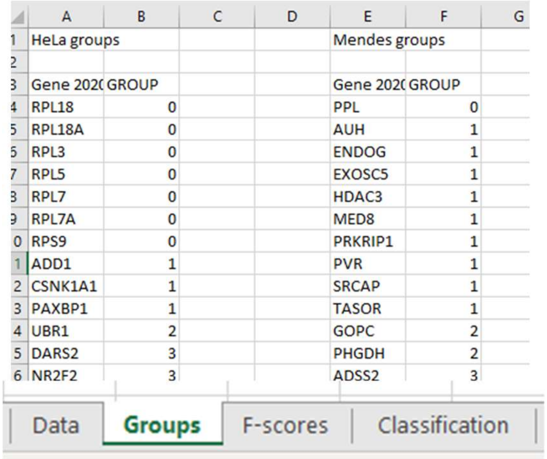

	A	B	C	D	E	F	G
1	HeLa groups				Mendes groups		
2							
3	Gene 202(	GROUP			Gene 202(	GROUP	
4	RPL18	0			PPL	0	
5	RPL18A	0			AUH	1	
5	RPL3	0			ENDOG	1	
7	RPL5	0			EXOSC5	1	
3	RPL7	0			HDAC3	1	
9	RPL7A	0			MED8	1	
0	RPS9	0			PRKRIP1	1	
1	ADD1	1			PVR	1	
2	CSNK1A1	1			SRCAP	1	
3	PAXBP1	1			TASOR	1	
4	UBR1	2			GOPC	2	
5	DARS2	3			PHGDH	2	
6	NR2F2	3			ADSS2	3	

Data | Groups | F-scores | Classification

The kgg output files from Cluster 3.0 contain protein identifiers and group assignment. The lists are copied into the «Groups» worksheet in the Analysis Workbook

7

Quick comparison of two datasets

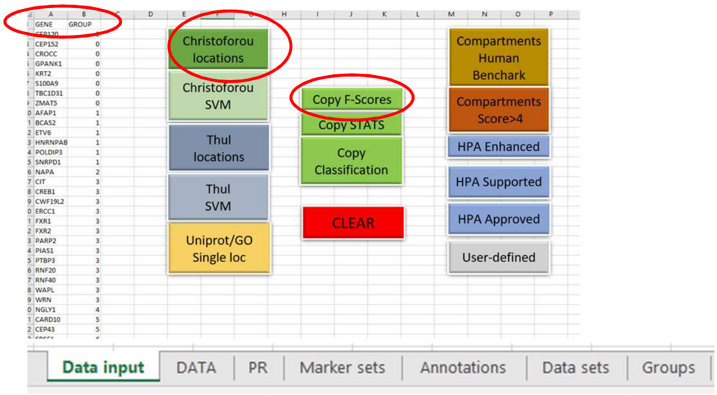

The clustering result for a given dataset is pasted into cell A1 in the Data input sheet in MetaMass
Click a button to select a marker set.
Click «Copy F-scores» and paste into the corresponding column in the F-score worksheet in the Analysis Workbook (see next page).
Repeat with all marker sets.

8

F-score bar graph

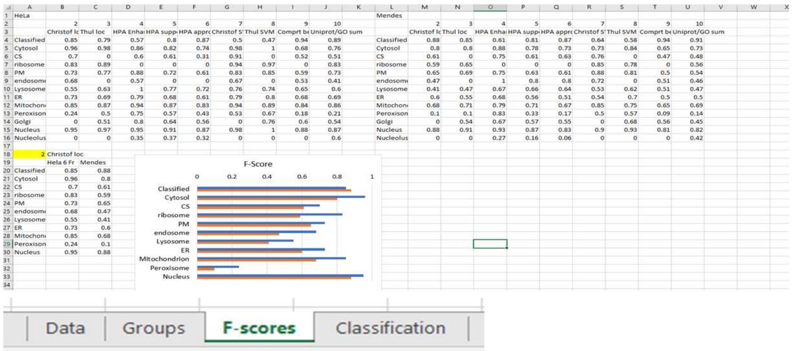

Paste F-scores (paste special: values) for each markerset set under the corresponding header in the «F-scores» sheet in the Analysis Workbook. When the operation is completed for both datasets, differences can be visualized using the bargraph in the F-scores sheet.
Enter numbers for marker sets in cell A18 (yellow) to select markerset for the bar graph
Typically, markersets from mass spectrometry data yield higher F-scores for subcellular proteomics data than do annotations from Uniprot, The Human Protein Atlas and the Compartments Database.

9

Classification: Assigned locations for individual proteins

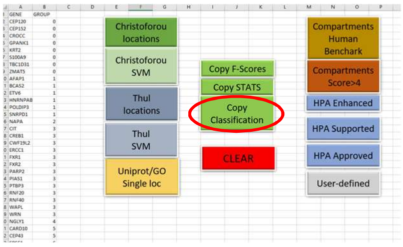

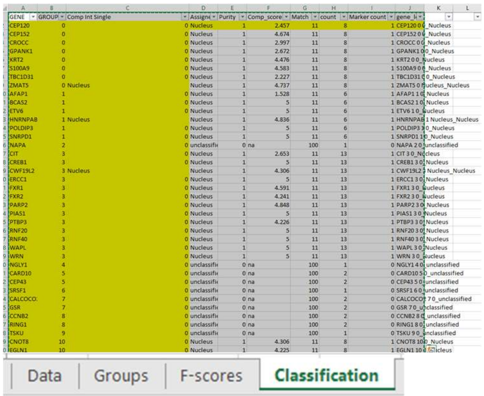

Use the Copy Classification button copy the list of assigned locations for each protein.
Paste into the Classification sheet in the Data Analysis Workbook

10

Sort the classification for each dataset to generate a heatmap with a consistent pattern

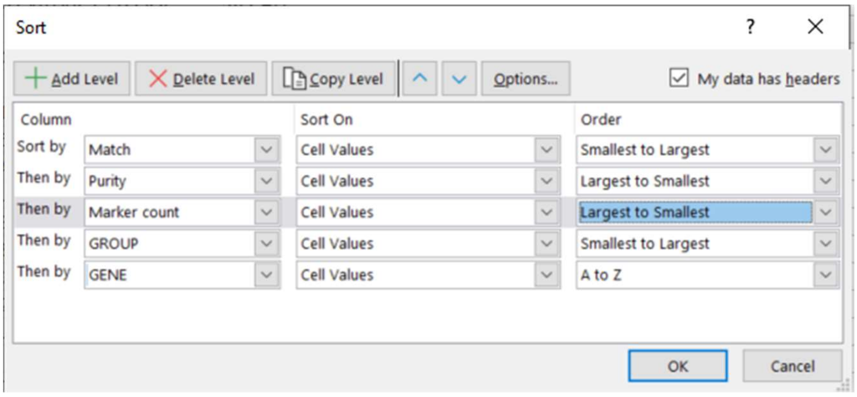

Match: numerical alias for location 1= cytosol, 12= nucleolus
Purity: fraction of markers in group corresponding to assigned location
Marker count: Number of markers in the group

11

Add a column with heatmap ordering for each of the two datasets

2	GENE	GROUP	Christoforou Assigi	Assigned	Purity	Comp_scc	Match	count	Marker co	gene_loc	HeLa Heatmap
3	ARHGDIA	507	Cytosol	CYTOSOL	1	4.706	1	22	16	ARHGDIA 507 Cytosol_CYTOSOL	1
4	ATIC	507	Cytosol	CYTOSOL	1	4.746	1	22	16	ATIC 507 Cytosol_CYTOSOL	2
5	BLVRA	507	0	CYTOSOL	1	4.715	1	22	16	BLVRA 507 0_CYTOSOL	3
5	CNDP2	507	Cytosol	CYTOSOL	1	4.578	1	22	16	CNDP2 507 Cytosol_CYTOSOL	4
7	ENO1	507	Cytosol	CYTOSOL	1	4.87	1	22	16	ENO1 507 Cytosol_CYTOSOL	5
3	FDPS	507	0	CYTOSOL	1	4.642	1	22	16	FDPS 507 0_CYTOSOL	6
9	FKBP1A	507	Cytosol	CYTOSOL	1	4.729	1	22	16	FKBP1A 507 Cytosol_CYTOSOL	7
0	GPI	507	Cytosol	CYTOSOL	1	4.874	1	22	16	GPI 507 Cytosol_CYTOSOL	8
1	GSTP1	507	Cytosol	CYTOSOL	1	4.854	1	22	16	GSTP1 507 Cytosol_CYTOSOL	9
2	HPRT1	507	0	CYTOSOL	1	4.836	1	22	16	HPRT1 507 0_CYTOSOL	10
3	NUDCD2	507	Cytosol	CYTOSOL	1	4.601	1	22	16	NUDCD2 507 Cytosol_CYTOSOL	11
4	NUDT5	507	Cytosol	CYTOSOL	1	4.36	1	22	16	NUDT5 507 Cytosol_CYTOSOL	12
5	OSTF1	507	0	CYTOSOL	1	3.808	1	22	16	OSTF1 507 0_CYTOSOL	13

GENE	GROUP	Christofor	Assigned	Purity	Comp_scc	Match	count	Marker co	gene_loc	Mendes Heatmap
CDC37	731	Cytosol	CYTOSOL	1	4.748	1	8	6	CDC37 731 Cytosol_CYTOSOL	1
DARS1	731	0	CYTOSOL	1	5	1	8	6	DARS1 731 0_CYTOSOL	2
ENO1	731	Cytosol	CYTOSOL	1	4.87	1	8	6	ENO1 731 Cytosol_CYTOSOL	3
PPP5C	731	Cytosol	CYTOSOL	1	4.787	1	8	6	PPP5C 731 Cytosol_CYTOSOL	4
PSMC3	731	cytosol	CYTOSOL	1	4.772	1	8	6	PSMC3 731 cytosol_CYTOSOL	5
PSMD5	731	cytosol	CYTOSOL	1	4.693	1	8	6	PSMD5 731 cytosol_CYTOSOL	6
TRMT5	731	0	CYTOSOL	1	2.581	1	8	6	TRMT5 731 0_CYTOSOL	7
TUBA4A	731	Cytosol	CYTOSOL	1	4.684	1	8	6	TUBA4A 731 Cytosol_CYTOSOL	8
ATP6V1F	88	0	CYTOSOL	1	4.575	1	8	4	ATP6V1F 88 0_CYTOSOL	9

12

Transfer heatmap ordering and locations to Datasheet

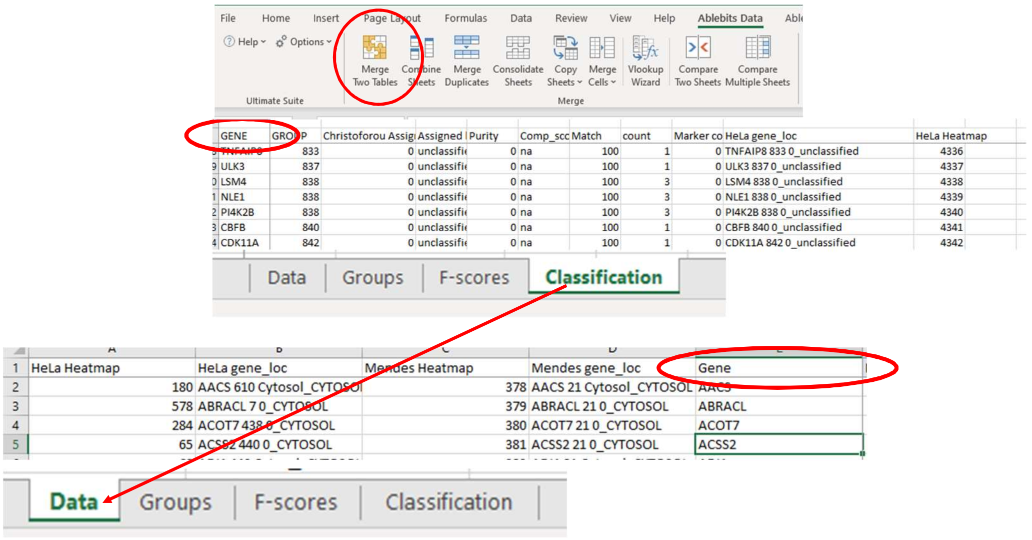

Add new columns to the datasheet and copy headers for heatmap ordering and protein locations from the Classification sheet. The AbleBits Excel Plugin Merge Two Tables is highly recommended for easy transfer of data between spreadsheets using one or more column(s) as common reference(s).

13

Use heatmaps to visualize final ordering of proteins

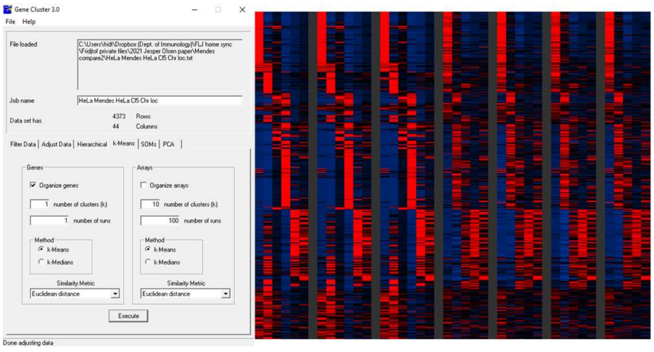

The proteins in the «Data» worksheet are sorted according to the ordering in the heatmaps. A new text file is saved and opened in Cluster 3.0. By choosing a single group for k-means clustering, the ordering in the heatmap will be the same as in the Excel spreadsheet.

14

Statistics

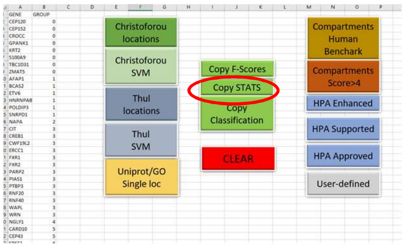

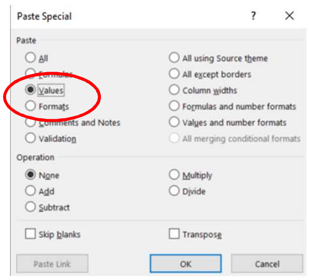

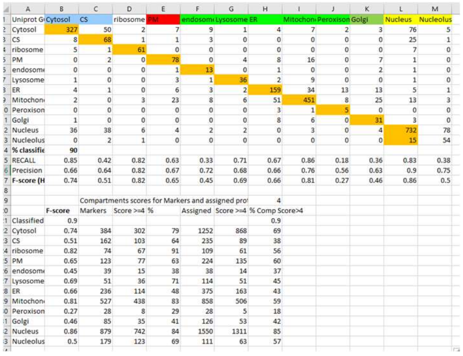

For more detailed analysis, use the Copy STATS button to copy statistics for recall and precision of the markers within a given set.
Open a new Excel Spreadsheet, select «paste special» Values and then Formats.
Click a cell in MetaMass II to inspect the formulas used to calculate recall, precision and F-score (harmonic mean of recall and precision).

15

Classification Method:

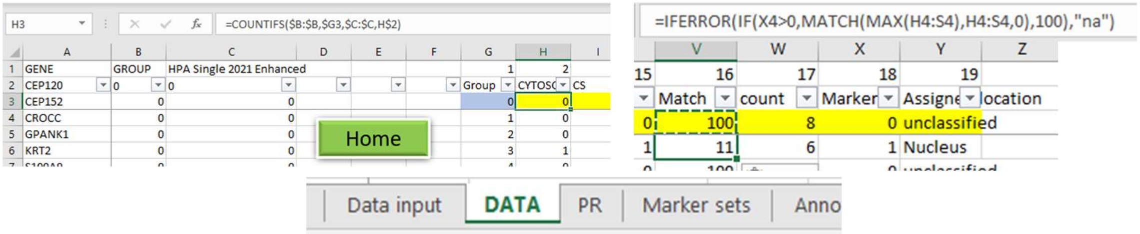

The algorithm counts markers for all subcellular locations within all groups. All proteins in the same group are assigned to the location with the highest marker count. Thus, if there are three markers for the nucleus and two for the cytosol, all are assigned to the nucleus. The purity is, however only 0.6; 3/(3+2), and the total number of markers in the group is 3. Higher purity and higher marker counts indicate higher precision for the assigned locations.

The classification is based on standard Excel functions. Click yellow cells to inspect them.

16

Annotations:

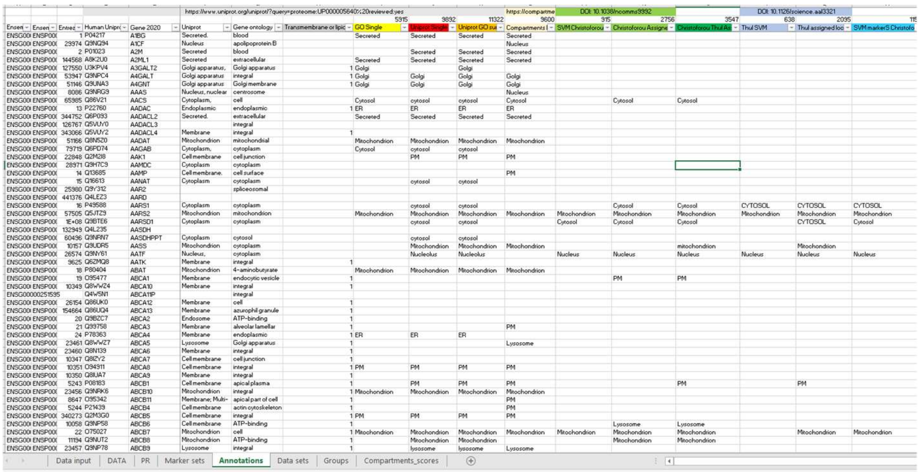

The annotations sheet in MetaMass contains annotations on the subcellular location of proteins from indicated sources. The marker sets were generated by filtering on single locations in full annotations.

Scores from the Compartments database serve as an independent reference

	F-score	Markers	Score >=4	%	Assigned	Score >=4	% Comp Score>4
Classified	0.9						0.9
Cytosol	0.74	384	302	79	1252	868	69
CS	0.51	162	103	64	235	89	38
ribosome	0.82	74	67	91	109	61	56
PM	0.65	123	77	63	224	135	60
endosome	0.45	39	15	38	38	14	37
Lysosome	0.69	51	36	71	114	51	45
ER	0.66	236	114	48	375	163	43
Mitochondrion	0.81	527	438	83	858	506	59
Peroxisome	0.27	28	8	29	28	5	18
Golgi	0.46	85	35	41	126	53	42
Nucleus	0.86	879	742	84	1550	1311	85
Nucleolus	0.5	179	123	69	111	63	57

MetaMass also classifies the assigned locations on basis of their fit with annotations in the Compartments Database. (https://compartments.jensenlab.org/Search, https://doi.org/10.1093/database/bau012) Annotations in the Compartments Database are not based on mass spectrometry data and therefore serve as an independent reference.

The spreadsheet returns the percentage of assigned proteins with a Compartments Database score higher than 4 (max= 5).

Supplementary References

1. Thul, P. J. *et al.* A subcellular map of the human proteome. *Science (80-.).* **356**, eaal3321 (2017).
2. Hornbeck, P. V. *et al.* PhosphoSitePlus, 2014: Mutations, PTMs and recalibrations. *Nucleic Acids Res.* **43**, D512–D520 (2015).
3. Christoforou, A. *et al.* A draft map of the mouse pluripotent stem cell spatial proteome. *Nat. Commun.* **7**, 8992 (2016).
4. Geladaki, A. *et al.* Combining LOPIT with differential ultracentrifugation for high-resolution spatial proteomics. *Nat. Commun.* **10**, 331 (2019).
5. Orre, L. M. *et al.* SubCellBarCode: Proteome-wide Mapping of Protein Localization and Relocalization. *Mol. Cell* **73**, 166-182.e7 (2019).
6. Krahmer, N. *et al.* Organellar Proteomics and Phospho-Proteomics Reveal Subcellular Reorganization in Diet-Induced Hepatic Steatosis. *Dev. Cell* **47**, 205-221.e7 (2018).
7. Crook, O. M. *et al.* A semi-supervised Bayesian approach for simultaneous protein sub-cellular localisation assignment and novelty detection. *PLoS Comput. Biol.* **16**, (2020).
