# Supplementary material for: Spatial-proteomics reveals phospho-signaling dynamics at subcellular resolution
Source: Nat Commun. 2021 Dec 7;12:7113. doi: 10.1038/s41467-021-27398-y (PMC8651693; doi:10.1038/s41467-021-27398-y)
Supplement: Supplementary file 3 — Description of Additional Supplementary Files [file 41467_2021_27398_MOESM3_ESM.pdf]

File Name: Supplementary Data 1. Results from Proteome HeLa Subcellular fractionation in control conditions.

**1A**	Raw intensity results at Protein Groups level from Spectronaut (V14) search for HeLa samples (in Control conditions)
**1B**	Log2 transformed intensity results at Protein Groups level. Data was normalized, filtered by missing values and imputed.
**1C**	Scaled intensities (from 0 to 1) for Proteins Groups of HeLa samples by replicate. It includes a categorical column ("hsap_location_marker") with the subcellular location markers used in the manuscript.
**1D**	Gene Ontology Cellular Component results for proteins enriched in each Fraction. Significance of the GO enrichment (over-representation analysis using hypergeometric test) is indicated by the "false discovery rate" column (multiple testing correction by BH).
**1E**	Gene Ontology Biological Process results for proteins enriched in Fraction 2. Significance of the GO enrichment (over-representation analysis using hypergeometric test) is indicated by the "false discovery rate" column (multiple testing correction by BH).
**1F**	Gene Ontology Cellular Component results for proteins enriched in Fraction 2. Significance of the GO enrichment (over-representation analysis using hypergeometric test) is indicated by the "false discovery rate" column (multiple testing correction by BH).

File Name: Supplementary Data 2. Results from Phospho-Proteome HeLa Subcellular fractionation in control conditions.

**2A**	Collapsed data to Phospho-sites using PTM results from Spectronaut V14
**2B**	Log2 transformed intensity results at Phospho-site level. Data was normalized, filtered by missing values and imputed.
**2C**	Scaled intensities (from 0 to 1) for Phospho-sites of HeLa samples by replicate. It includes a categorical column ("hsap_location_marker") with the subcellular location markers used in the manuscript.

File Name: Supplementary Data 3. Results from Proteome U2OS Subcellular fractionation in control conditions.

**3A**	Raw intensity results at Protein Groups level from Spectronaut (V14) search for U2OS samples (in Control conditions)
**3B**	Log2 transformed intensity results at Protein Groups level. Data was normalized, filtered by missing values and imputed.
**3C**	Scaled intensities (from 0 to 1) for Proteins Groups of U2OS samples by replicate. It includes a categorical column ("hsap_location_marker") with the subcellular location markers used in the manuscript.

File Name: Supplementary Data 4.

Excel Macro with MetaMass II tool.

File Name: Supplementary Data 5. Results from Proteome and Phospho-proteome of HeLa cells subcellular fractionation in response to EGF stimulation.

**5A**	Raw intensity results at Protein Groups level from Spectronaut (V14) search for HeLa samples treated with EGF at all time points.
**5B**	Log2 transformed intensity results at Protein Groups level for HeLa+EGF experiment. Data was normalized, filtered by missing values and imputed.
**5C**	Collapsed data to Phospho-sites using PTM results from Spectronaut (V14) search for HeLa samples treated with EGF at all time points.
**5D**	Log2 transformed intensity results at Phospho-site level for HeLa + EGF experiment. Data was normalized, filtered by missing values and imputed.

File Name: Supplementary Data 6.

Differential expression results for the comparisons of each time point of EGF treatment VS Control condition, for each subcellular fractions. Statistical analysis was performed using moderated t-test (limma, two-sided). Multiple comparisons was adjusted using Benjaminin-Hochberg.

File Name: Supplementary Data 7.

Differential expression result at phosphosite level for the comparisons of each time point of EGF treatment VS Control condition, for each subcellular fractions. Statistical analysis was performed using moderated t-test (limma, two-sided). Multiple comparisons was adjusted using Benjaminin-Hochberg.

File Name: Supplementary Data 8. Results from Proteome and Phospho-proteome of mouse kidneys subcellular fractionation in response to EGF stimulation.

**8A**	Raw intensity results at Protein Groups level from Spectronaut (V14) search for Mouse kidney samples treated with EGF at all time points.
**8B**	Log2 transformed intensity results at Protein Groups level for Mouse kidney+EGF experiment. Data was normalized, filtered by missing values and imputed.
**8C**	Collapsed data to Phospho-sites using PTM results from Spectronaut (V14) search for Mouse kidney samples treated with EGF at all time points.
**8D**	Log2 transformed intensity results at Phospho-site level for Mouse kidney + EGF experiment. Data was normalized, filtered by missing values and imputed.

File Name: Supplementary Data 9. Results from Proteome and Phospho-proteome of mouse liver subcellular fractionation in response to EGF stimulation.

**9A**	Raw intensity results at Protein Groups level from Spectronaut (V14) search for Mouse Liver samples treated with EGF at all time points.
**9B**	Log2 transformed intensity results at Protein Groups level for Mouse Liver+EGF experiment. Data was normalized, filtered by missing values and imputed.
**9C**	Collapsed data to Phospho-sites using PTM results from Spectronaut (V14) search for Mouse Liver samples treated with EGF at all time points.
**9D**	Log2 transformed intensity results at Phospho-site level for Mouse Liver + EGF experiment. Data was normalized, filtered by missing values and imputed.

File Name: Supplementary Data 10. Results from Proteome and Phospho-proteome of U2OS cells subcellular fractionation in response to osmotic stress.

**10A**	Raw intensity results at Protein Groups level from Spectronaut (V14) search for U2OS samples treated with Sorbitol for 1 hour and after realease at 30 minutes, 3 hours and 24 hours.
**10B**	Log2 transformed intensity results at Protein Groups level for U2OS+sorbitol experiment. Data was normalized, filtered by missing values and imputed.
**10C**	Collapsed data to Phospho-sites using PTM results from Spectronaut (V14) search for U2OS samples treated with sorbitol at all time points.
**10D**	Log2 transformed intensity results at Phospho-site level for U2OS + sorbitol experiment. Data was normalized, filtered by missing values and imputed.

File Name: Supplementary Data 11.

Differential expression results for the comparisons of each time point of Sorbitol/release treatment VS Control condition, for each subcellular fractions. Statistical analysis was performed using moderated t-test (limma, two-sided). Multiple comparisons was adjusted using Benjaminin-Hochberg.

File Name: Supplementary Data 12.

Differential expression results at phospho-site level for the comparisons of each time point of Sorbitol/release treatment VS Control condition, for each subcellular fractions. Statistical analysis was performed using moderated t-test (limma, two-sided). Multiple comparisons was adjusted using Benjaminin-Hochberg.

File Name: Supplementary Data 13. Results from Proteome and Phospho-proteome of mouse muscle subcellular fractionation in response to contraction.

**13A**	Raw intensity results at Protein Groups level from Spectronaut (V14) search for Mouse muscle samples in rest and stretching conditions.
**13B**	Log2 transformed intensity results at Protein Groups level for Mouse muscle samples in rest and stretching conditions. Data was normalized, filtered by missing values and imputed.
**13C**	Collapsed data to Phospho-sites using PTM results from Spectronaut (V14) search for Mouse muscle samples in rest and stretching conditions.
**13D**	Log2 transformed intensity results at Phospho-site level forMouse muscle samples in rest and stretching conditions. Data was normalized, filtered by missing values and imputed.
