# Supplementary material for: Spatial-proteomics reveals phospho-signaling dynamics at subcellular resolution
Source: Nat Commun. 2021 Dec 7;12:7113. doi: 10.1038/s41467-021-27398-y (PMC8651693; doi:10.1038/s41467-021-27398-y)
Supplement: Supplementary file 17 — Reporting Summary [file 41467_2021_27398_MOESM17_ESM.pdf]

nature research

Corresponding author(s): Jesper V. Olsen

Last updated by author(s): Oct 29, 2021

Reporting Summary

Nature Research wishes to improve the reproducibility of the work that we publish. This form provides structure for consistency and transparency in reporting. For further information on Nature Research policies, see our Editorial Policies and the Editorial Policy Checklist.

Statistics

For all statistical analyses, confirm that the following items are present in the figure legend, table legend, main text, or Methods section.

n/a	Confirmed	
[ ]	[x]	The exact sample size (*n*) for each experimental group/condition, given as a discrete number and unit of measurement
[ ]	[x]	A statement on whether measurements were taken from distinct samples or whether the same sample was measured repeatedly
[ ]	[x]	The statistical test(s) used AND whether they are one- or two-sided<br>*Only common tests should be described solely by name; describe more complex techniques in the Methods section.*
[ ]	[x]	A description of all covariates tested
[ ]	[x]	A description of any assumptions or corrections, such as tests of normality and adjustment for multiple comparisons
[ ]	[x]	A full description of the statistical parameters including central tendency (e.g. means) or other basic estimates (e.g. regression coefficient) AND variation (e.g. standard deviation) or associated estimates of uncertainty (e.g. confidence intervals)
[ ]	[x]	For null hypothesis testing, the test statistic (e.g. *F*, *t*, *r*) with confidence intervals, effect sizes, degrees of freedom and *P* value noted<br>*Give P values as exact values whenever suitable.*
[x]	[ ]	For Bayesian analysis, information on the choice of priors and Markov chain Monte Carlo settings
[x]	[ ]	For hierarchical and complex designs, identification of the appropriate level for tests and full reporting of outcomes
[x]	[ ]	Estimates of effect sizes (e.g. Cohen's *d*, Pearson's *r*), indicating how they were calculated

Our web collection on statistics for biologists contains articles on many of the points above.

Software and code

Policy information about availability of computer code

Data collection	Mass spectrometry data was acquired using the Orbitrap Exploris 480 MS, using Xcalibur (Tune software version: 1.1 Thermo)
Data analysis	Proteomics data was analysed using Spectronaut (v14 and v15) and further processed using Perseus (v1.6.5.0) and custom R (v3.6.0) code (DAPAR package 1.18.3, Prostar package 1.18.4, imp4p package 0.8, limma package v3.42).<br>PTM collapse plugin requires Perseus and R (minimum version 3.6.0) to run and it is available at github.com/AlexHgO/Perseus_Plugin_Peptide_Collapse.<br>Northern blotting membranes were analyzed using Fiji software (v1.53c)

For manuscripts utilizing custom algorithms or software that are central to the research but not yet described in published literature, software must be made available to editors and reviewers. We strongly encourage code deposition in a community repository (e.g. GitHub). See the Nature Research guidelines for submitting code & software for further information.

Data

Policy information about availability of data

All manuscripts must include a data availability statement. This statement should provide the following information, where applicable:

- Accession codes, unique identifiers, or web links for publicly available datasets
- A list of figures that have associated raw data
- A description of any restrictions on data availability

The mass spectrometry proteomics data have been deposited to the ProteomeXchange Consortium via the PRIDE partner repository with the dataset identifier PXD023690.

Proteomics data was analysed using: human database (Uniprot reference proteome 2019 release, 21074 entries) or mouse database (Uniprot reference proteome 2019 release, 22286 entries)

Field-specific reporting

Please select the one below that is the best fit for your research. If you are not sure, read the appropriate sections before making your selection.

[x] Life sciences [ ] Behavioural & social sciences [ ] Ecological, evolutionary & environmental sciences

For a reference copy of the document with all sections, see nature.com/documents/nr-reporting-summary-flat.pdf

Life sciences study design

All studies must disclose on these points even when the disclosure is negative.

Sample size	No sample size calculation was performed. Number of replicates was chosen based on previous expertise to obtain enough statistical power. In all experiments, except for the muscle contraction, four biological replicates: either four cell dishes or four mice per condition for liver and kidney experiments, and three mice for muscle experiments were employed. The number of replicates was chosen to show reproducibility during the sample preparation of the subcellular fractions using the protocol described in the paper.<br>Live imaging experiments were performed always in three technical replicates.
Data exclusions	No data were excluded from the analysis.
Replication	All experiments were performed using replicates as stated in the sample size section. All samples for the same experiment were processed in parallel. The reproducibility of the replicates is described in the text, where we showed good correlation between independent subcellular preparations, at cell and tissue level.
Randomization	Allocation of cells and mice to experimental or control group was random. Randomization in the rest of the experiments is not relevant in this study since all samples from each experiment are analyzed by replicate group and during the same acquisition period, to minimize technical variability.
Blinding	Researchers were not blinded to perform the experiments reported in this work as to keep a strict overview of sample preparation, LC-MS/MS runs and data analysis.

Reporting for specific materials, systems and methods

We require information from authors about some types of materials, experimental systems and methods used in many studies. Here, indicate whether each material, system or method listed is relevant to your study. If you are not sure if a list item applies to your research, read the appropriate section before selecting a response.

Materials & experimental systems

n/a	Involved in the study	
[ ]	[x]	Antibodies
[ ]	[x]	Eukaryotic cell lines
[x]	[ ]	Palaeontology and archaeology
[ ]	[x]	Animals and other organisms
[x]	[ ]	Human research participants
[x]	[ ]	Clinical data
[x]	[ ]	Dual use research of concern

Methods

n/a	Involved in the study	
[x]	[ ]	ChIP-seq
[x]	[ ]	Flow cytometry
[x]	[ ]	MRI-based neuroimaging

Antibodies

Antibodies used	keima anti-mouse (MBL M126-3M, 1:200); fibrillarin anti-rabbit (abcam ab5821, 1:200); Alexa Fluor rabbit-568 (Thermo Fisher A10042, 1:1000) and Alexa Fluor mouse-488 (Thermo Fisher A11029, 1:1000).
Validation	All antibodies used were commercial and validated by supplier.<br>Keima anti-mouse antibody has been previously used in the following publications: Cell Rep. 24, 2404-2417.e8 (2018) and Invest Ophthalmol Vis Sci. 60, 3625-3635 (2019). Fibrillarin anti-rabbit antibody is highly specific towards the target protein, the manufacturer states "This antibody detects a band at close to 34kDa in all species tested. The band can be completely blocked with the immuising peptide in all cases - this is very strong evidence that the antibody is recognising fibrillarin." It has been also used in multiple publications, inlcuding: PLoS Genet 17:e1009362 (2021) or Chromosoma 129:121-139 (2020).

Eukaryotic cell lines

Policy information about cell lines

Cell line source(s)	HeLa, U2OS and HEK293 were acquired from ATCC.<br>TIG3 cells were a gift from Anders Lund's lab at Biotech Research & Innovation Center, University of Copenhagen.
Authentication	Cell lines were authenticated by STR profiling using the ATCC authentication service according to manufacturers' instructions.
Mycoplasma contamination	Cultures tested negative for mycoplasma.
Commonly misidentified lines<br>(See ICLAC register)	No commonly misidentified cell lines were employed in this study.

Animals and other organisms

Policy information about studies involving animals; ARRIVE guidelines recommended for reporting animal research

Laboratory animals	Littermate male C57BL/6JRj mice obtained from Janvier Labs (Le Genest-Saint-Isle, France) at six weeks of age.
Wild animals	No wild animals were used in this study.
Field-collected samples	No field collected samples were used in this study.
Ethics oversight	Mice experiments carried out in this study were performed according to the guidelines of the Danish Animal Welfare Act and the Directive 2010/63/EU for the protection of animals used for scientific purpose. The study was approved by the Institutional Animal Care and Use Committee of the University of Copenhagen and the Animal Experiments Inspectorate, Ministry of Environment and Food, Denmark (License number 2020-15-0201-00508 and 2019-15-0201-01659) and the Institutional Animal Care and Use committee of the University of Copenhagen (Project number P20-372 and P19-342).

Note that full information on the approval of the study protocol must also be provided in the manuscript.
